# Unusual Etiology of Budd‐Chiari Syndrome in an Adolescent: A Case of Combined Thrombophilic Disorder

**DOI:** 10.1002/ccr3.71606

**Published:** 2025-12-11

**Authors:** Santosh Sah, Rumaisa Riaz, Afsheen Khan, Tularam Yadav, Kirshan Lal, Hussain Haider Shah

**Affiliations:** ^1^ Internal Medicine Dow University of Health Sciences (DUHS) Karachi Pakistan; ^2^ Primary Healthcare Centre (PHCC) Jhorahat Gramthan Koshi Nepal

**Keywords:** adolescent, Budd‐Chiari syndrome, hepatomegaly, hypercoagulable state, inherited thrombophilia

## Abstract

Budd‐Chiari syndrome (BCS) is an uncommon but potentially life‐threatening hepatic vascular disorder resulting from obstruction of the hepatic venous outflow. While it predominantly affects adults, pediatric and adolescent presentations are rare, particularly those associated with inherited thrombophilia. Early diagnosis and appropriate intervention are essential to prevent complications such as portal hypertension, hepatic ischemia, and liver failure. We describe the case of a 15‐year‐old female who presented with progressive abdominal distension, right upper quadrant pain, and bilateral lower limb edema. Imaging studies, including ultrasound and contrast‐enhanced CT, revealed hepatic venous obstruction with thrombus extension into the portal, superior mesenteric and splenic veins, and gross ascites. Laboratory findings indicated elevated inflammatory markers and impaired liver function. A detailed thrombophilia workup demonstrated deficiencies in protein C and antithrombin III and a Factor V Leiden mutation, confirming a hypercoagulable state as the underlying etiology. The patient was diagnosed with subacute Budd‐Chiari Syndrome secondary to inherited thrombophilia. She was initiated on anticoagulation therapy with warfarin, later transitioned to rivaroxaban, and managed with diuretics and dietary modifications. Clinical improvement was achieved, and the patient was referred to a liver transplant center for ongoing evaluation.

## Key Clinical Message

- This case underscores the importance of maintaining a high index of suspicion for thrombophilic disorders in young patients presenting with nonspecific abdominal symptoms and imaging findings suggestive of hepatic vascular compromise.
- Unexplained portal hypertension with preserved liver function should prompt an evaluation for noncirrhotic causes, particularly an underlying hypercoagulable state.
- Early recognition, targeted anticoagulation, and timely referral can significantly alter the disease trajectory and improve long‐term outcomes.

## 1 Introduction

Budd‐Chiari Syndrome (BCS) is a rare hepatic vascular disorder resulting from obstruction of the hepatic venous outflow tract, from the hepatic vein (HV) to the entry of the inferior vena cava (IVC) into the heart [1]. The global incidence of BCS remains low with an estimated one new case per million individuals annually, with a higher prevalence reported in the Asian population [2].

BCS presents in two forms: acute, characterized by a sudden hepatic outflow obstruction, and subacute or chronic, resulting from previous venous obstruction [3]. Clinical manifestations vary widely, from asymptomatic cases to fulminant liver failure, depending on the site of obstruction and extent of liver involvement. Studies show ascites as the most common symptom, followed by hepatomegaly, esophageal varices, and gastrointestinal bleeding [4, 5].

Thrombophilia is a major predisposing risk factor for BCS, encompassing both inherited causes such as protein C, protein S, antithrombin III deficiency, Factor V Leiden mutation, and acquired conditions including antiphospholipid syndrome, hypercoagulable states, and myeloproliferative disorder [6]. Systemic disorders like inflammatory bowel disease, Behcet's disease, and other inflammatory intra‐abdominal lesions can cause BCS in adults, but it is a rare implication in the pediatric population [7]. Recent studies report an identifiable etiology in approximately 80%–84% of adult cases [8]. In adolescents, BCS remains exceptionally rare, and subacute presentations secondary to thrombophilia are even less frequently reported, emphasizing the need for high clinical suspicion in young patients presenting with unexplained abdominal vascular symptoms [9]. The initial treatment is a DOAC (apixaban, rivaroxaban), stepping up to combined DOAC + LMWH for breakthrough thrombosis, with liver transplantation as the final option for refractory cases [10].

This report presents the case of a female patient with subacute Budd‐Chiari syndrome secondary to thrombophilia. The case underscores the critical importance of investigating underlying coagulation disorders in young patients with abdominal symptoms and radiological evidence of hepatic vascular compromise to mitigate long‐term hepatic sequelae.

## 2 Case History/Examination

A 15‐year‐old female with no comorbidity presented to the outpatient department of Internal Medicine, with abdominal distension and pain along with low‐grade intermittent fever for 1 month. Pain was localized to the right upper quadrant, intermittent, gradual onset with aching in character. She also had complaints of shortness of breath on exertion, with dizziness, palpitations, easy fatigability, decreased appetite, constipation, and decreased urinary frequency along with bilateral lower limb swelling while past history was insignificant. Family history was unremarkable for any clotting disorder. The patient denied the use of contraceptive pills. On physical examination, she was pale but had no jaundice with a distended abdomen, tenderness in the right upper quadrant, a palpable liver 2 cm below the costal margin, shifting dullness and bilateral limb swelling, while the rest of the systemic examinations were unremarkable. Vital parameters were recorded as follows: Blood pressure (BP), 90/60 mmHg with no postural drop; oxygen saturation (SpO_2_), 94% (without oxygen support); heart rate (HR), 95 beats/min; respiratory rate (RR), 16/min; and temperature of 98°F.

## 3 Methods (Differential Diagnosis, Investigations, and Treatment)

Abdominal tuberculosis, chronic liver disease and Budd‐Chiari syndrome were included in the differential diagnosis. An abdominal ultrasound was performed, which revealed a liver with altered echotexture associated with impending thrombus in the portal vein, no splenomegaly, thick‐walled gallbladder, bulky pancreas and moderate to gross ascites. Initial investigations included complete blood counts, inflammatory markers such as C‐reactive protein (CRP), urine pregnancy test (UPT) and erythrocyte sedimentation rate (ESR), serum electrolytes, liver function tests (LFTs), coagulation profile, iron profile, urine detailed report (DR), and a chest x‐ray (Table 1).

**TABLE 1:** Pathological values of the laboratory tests performed in the emergency department.

Laboratory parameter	Conventional units	Value (in ED)	Reference Range
WBC	× 10^3^/μL	9.9	4–10
HGB	g/dL	7.9	12–15
SAAG	g/dL	2.1	
ALT	U/L	59	14–59
BT (total bilirubin)	mg/dL	1.7	0.2–1
BD (direct bilirubin)	mg/dL	0.9	0.0–0.2
AG ratio		1.03	
Creatinine	mg/dL	0.5	0.55–1.02
FAL (alkaline phosphatase)	U/L	110	35–105
Ferritin	ng/mL	16	15–150
CRP	mg/L	15.3	0.0–5.0
UPT	Negative		
INR		1.47	0.8–1.2
aPTT	Seconds	24.1	21.6–28.7

Abbreviations: AG, albumin globulin; ALT, alanine aminotransferase; aPTT, activated partial thromboplastin time; BD, direct bilirubin; BT, total bilirubin; CRP, C‐reactive protein; FAL, alkaline phosphatase; INR, international normalized ratio; HGB, hemoglobin; RBC, red blood cell; SAAG, serum‐ascites albumin gradient; UPT, urine pregnancy test.

**FIGURE 1:**
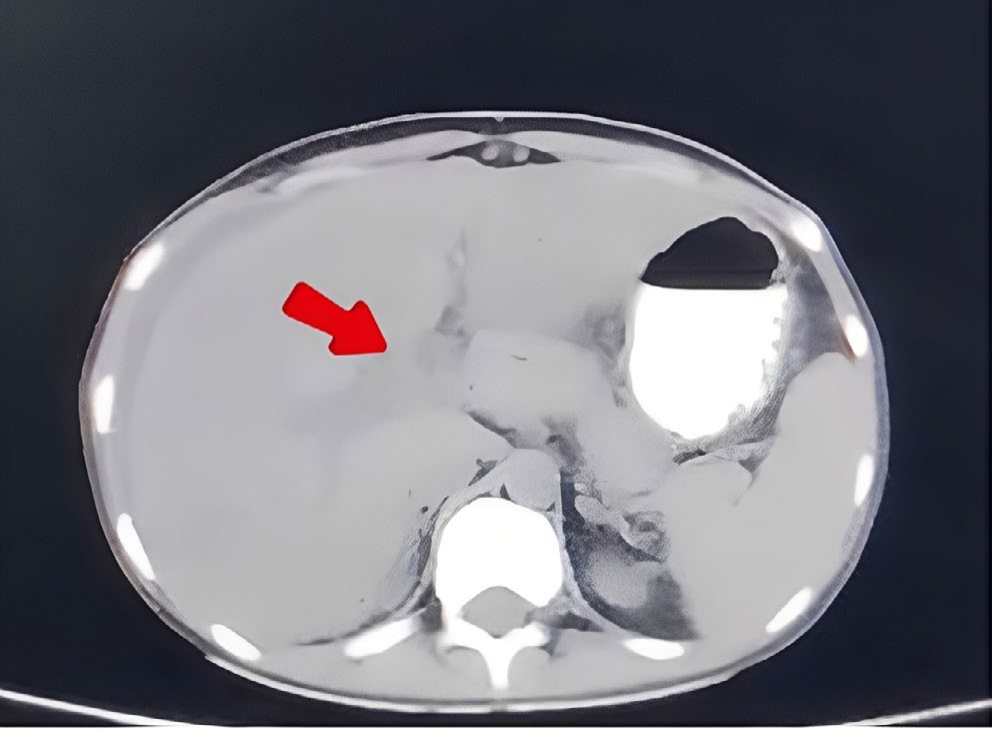
CT abdomen showing early enhancement of the central part of the liver and the caudate lobe around the inferior vena cava (IVC), with evidence of a filling defect in the main portal vein. The right, middle, and left hepatic veins are not visualized. Perihepatic free fluid is seen, suggestive of ascites.

Further workup included ascitic fluid DR, echocardiogram, antinuclear antibody (ANA) profile, extractable nuclear antigen (ENA) profile, and endoscopy which showed only mild antral gastritis. Pre and postcontrast‐enhanced computed tomography (CT scan) of the whole abdomen was carried out; images were obtained in multiple planes and viewed at appropriate window settings which revealed a normal‐sized liver measuring 16.5 cm and a portal vein of 8.3 mm. There was early enhancement of the caudate lobe and central liver around the inferior vena cava with delayed enhancement of the peripheral liver with accompanying central low density (Figure 1). The right, middle and left hepatic veins were not visualized in the Porto‐venous phase. No diffusely infiltrative process or focal mass was seen. There was no evidence of intrahepatic or extrahepatic biliary duct dilatation. A filling defect was seen in the main portal vein which extended up to the superior mesenteric as well as in the proximal and mid splenic vein. Gross ascites with diffuse mesenteric fat stranding were seen. Few subcentimetric lymph nodes were noted in the mesentery, the largest measuring 5.7 mm. The gallbladder, spleen, kidneys, adrenal glands, and urinary bladder were found to be normal. The findings were suggestive of acute Budd‐Chiari syndrome with ascites and portal vein thrombosis extending into the superior mesenteric as well as the splenic vein.

## 4 Conclusion and Results (Outcome and Follow‐Up)

The thrombophilia profile was done to determine the etiology which revealed low levels of protein C, Antithrombin III and Factor V Leiden, while Protein S and Lupus Anticoagulant were within the normal ranges (Table 2 shows the thrombophilia profile, a blood test assessing the risk of blood clots). Thus, the final diagnosis of subacute Budd‐Chiari syndrome secondary to Thrombophilia was made.

**TABLE 2:** This table shows the Thrombophilia profile, a comprehensive blood test used to evaluate an individual's risk for developing blood clots, known as thrombophilia.

Tests	Result (IU/dL)	Normal range (IU/dL)
Protein C	31.7	70–140
Protein S	72.9	60–130
Lupus anticoagulant	0.8	0.8–1.2
Antithrombin III	50.8	79–112
Factor V Leiden	0.81	0.86–1.10

The patient was admitted to the ward and started on the tablet Warfarin 5 mg. once daily with regular INR monitoring that improved her condition. She was discharged from the hospital with a prescription of tablet Rivaroxaban 10 mg once daily along with tablet Spironolactone 100 mg twice daily, tablet Furosemide 40 mg. once daily, folate supplements, counseled for salt restriction and referred to the Liver Transplant Center.

## 5 Discussion

Budd‐Chiari syndrome (BCS) is a rare condition in the pediatric population, characterized by obstruction of hepatic venous outflow. This blockage can occur at any point from the small hepatic venules up to the junction where the inferior vena cava (IVC) enters the right atrium. Clinical symptoms typically emerge when at least two hepatic veins are occluded, leading to increased sinusoidal pressure and decreased blood flow, which contribute to liver congestion and ischemia [11]. The site of obstruction, the number of veins involved, and the speed of occlusion all play critical roles in the clinical and pathological variation observed in BCS cases [12].

BCS is broadly categorized into primary and secondary types. Primary BCS is most commonly caused by thrombosis, particularly in individuals with prethrombotic lesions or underlying hypercoagulable states. Secondary BCS, on the other hand, arises from external compression or invasion of the hepatic outflow tract by tumors, such as hepatic or IVC malignancies and leiomyomas [13]. Although acquired conditions such as myeloproliferative disorders, antiphospholipid syndrome, and paroxysmal nocturnal hemoglobinuria (PNH) are less commonly seen in children, systemic disorders including celiac disease, Behçet's disease, sarcoidosis, immunoallergic vasculitis, and granulomatosis can also predispose individuals to BCS [14]. Infections, hydatid disease, and benign or malignant liver tumors can similarly lead to hepatic outflow obstruction [15].

In the patient discussed, a rare combination of multiple inherited thrombophilia conditions: Protein C deficiency, Antithrombin III deficiency, and Factor V Leiden mutation was identified, creating a significantly heightened risk for widespread thrombosis. In addition to hepatic vein involvement, thrombi were present in the portal and superior mesenteric and splenic veins, highlighting a severe hypercoagulable state.

BCS can present in different forms: fulminant, acute, subacute, and chronic. Fulminant cases involve the rapid onset of severe upper abdominal pain, jaundice, vomiting, ascites, and liver failure, with a high risk of mortality. Acute cases manifest with the quick development of ascites and hepatic necrosis, typically without the formation of venous collaterals. Subacute BCS, which was suspected in this patient, often has a slower and more insidious onset, allowing time for the development of collateral circulation, thereby minimizing liver necrosis and making it the most commonly observed form. Chronic cases may lead to caudate lobe hypertrophy, which can further compress the IVC and result in cirrhosis and ascites [16].

Diagnosis relies heavily on imaging. Conventional and color Doppler ultrasound may show altered hepatic flow patterns, intrahepatic collaterals, and ascites [17]. In this patient, ultrasound findings included altered liver echotexture, impending portal vein thrombus, bulky pancreas, moderate to gross ascites, and no splenomegaly. Doppler studies can detect decreased or reversed hepatic venous flow, subcapsular collaterals, and characteristic waveform changes. CT imaging is used to assess liver morphology and vascular changes, often revealing caudate lobe enlargement and arterial phase enhancement [9].

If left untreated, BCS carries a poor prognosis, with a 3‐year mortality rate of up to 90%. Treatment is typically stepwise, beginning with anticoagulation, followed by endovascular procedures such as angioplasty or stenting, and, if needed, transjugular intrahepatic portosystemic shunting (TIPS) [18]. However, TIPS can be challenging in children due to anatomical limitations and the risk of significant hemodynamic shifts [19]. Liver transplantation (LT) is considered the most definitive treatment in cases unresponsive to other therapies, and it is often the preferred option in pediatric patients [20]. While some children may respond to anticoagulation alone, long‐term management generally requires lifelong anticoagulation. Although interventional radiological approaches have shown success in adults, data in pediatric populations remain limited [21], making liver transplantation the most reliable option in severe pediatric cases.

## 6 Conclusion

This case highlights the importance of considering inherited thrombophilic disorders, such as deficiencies in protein C, protein S, and antithrombin III, in young patients presenting with features suggestive of Budd‐Chiari syndrome. Early recognition and appropriate intervention in the subacute phase can dramatically improve outcomes, prevent progression to chronic liver disease, and reduce the need for invasive procedures. Clinicians should maintain a high index of suspicion in pediatric and adolescent patients with unexplained abdominal pain, hepatomegaly, or ascites, especially in the context of known or suspected thrombophilic states.

## Author Contributions

**Santosh Sah:** conceptualization, data curation, investigation, methodology, project administration, resources, software, supervision, validation, visualization, writing – original draft, writing – review and editing. **Rumaisa Riaz:** conceptualization, data curation, investigation, methodology, project administration, resources, software, supervision, validation, visualization, writing – review and editing. **Afsheen Khan:** conceptualization, data curation, formal analysis, investigation, methodology, project administration, resources, software, supervision, validation, visualization, writing – original draft, writing – review and editing. **Tularam Yadav:** conceptualization, data curation, formal analysis, investigation, methodology, project administration, supervision, validation, visualization, writing – original draft, writing – review and editing. **Kirshan Lal:** conceptualization, data curation, investigation, methodology, project administration, resources, supervision, validation, visualization, writing – original draft, writing – review and editing. **Hussain Haider Shah:** conceptualization, data curation, investigation, project administration, resources, supervision, validation, visualization, writing – original draft, writing – review and editing.

## Funding

The authors have nothing to report.

## Consent

Written informed consent was obtained from the patient for the publication of this case report and images. A copy of the written consent is available for review by the Editor‐in‐Chief of this journal upon request.

## Conflicts of Interest

The authors declare no conflicts of interest.

## Data Availability

The datasets generated and analyzed during the current study are available from the corresponding author upon reasonable request. The data supporting the findings of this study have been de‐identified to ensure the participant's confidentiality and privacy.
